# Event-Camera-Based Defect Detection of High-Speed Rotating Propellers

**DOI:** 10.3390/s26154721

**Published:** 2026-07-25

**Authors:** Yosuke Ishikawa, Kenji Iwata, Yutaka Satoh

**Affiliations:** 1 National Institute of Advanced Industrial Science and Technology (AIST), Tsukuba 305-8560, Japan; kenji.iwata@aist.go.jp (K.I.); yu.satou@aist.go.jp (Y.S.); 2 Graduate School of Science and Technology, University of Tsukuba, Tsukuba 305-8573, Japan

**Keywords:** event camera, event-based vision, defect detection, defect progression monitoring

## Abstract

Defect detection in rotating propellers is critical because small defects can cause performance degradation, vibration, noise, and safety risks. Conventional frame-based cameras suffer from motion blur, whereas high-speed cameras often require costly equipment and controlled conditions. This study investigates an event-camera-based sensing framework that converts asynchronous luminance changes from high-speed rotating propellers into positive–negative (PN) composite event accumulation images and classifies normal and defective propellers using a convolutional neural network (CNN). Gradient-weighted Class Activation Mapping (Grad-CAM) visualizes class-discriminative regions and extracts candidate defect regions, while normalized cross-correlation generates event accumulation images at the same rotational phase for defect progression monitoring. Experiments with 65 mm three-blade propellers with approximately 1 mm artificial blade defects rotating at approximately 862 rpm showed that, in an exploratory accumulation-time analysis, 900 μs produced an image-level accuracy of 0.93; integrating images over one full rotation yielded no misclassifications under the present dataset. An additional experiment at 2610 rpm tracked defect progression using a quantitative indicator based on distance-transform pixel counts. These results support the feasibility of event-based sensing for defect detection, cropping candidate defect-related regions, and tracking an image-domain indicator associated with defect progression in high-speed rotating propellers under the tested conditions.

## 1. Introduction

Defect detection in rotating components used in manufacturing plants and industrial equipment is important for supporting equipment safety [1]. In particular, for high-speed rotating components such as propellers, fans, and turbine blades, even small defects or cracks in part of a blade can lead to reduced aerodynamic performance, increased vibration and noise, and, in severe cases, serious accidents caused by component failure. Therefore, sensing technologies that may support non-contact defect detection during operation, candidate-region estimation, and observation of defect-related changes are desirable. In recent years, defect detection methods based on machine vision, which combine frame-based cameras with image processing or deep learning, have been widely studied for the automation of visual inspection [2]. However, unlike ordinary visual inspection, a propeller rotating at high speed moves substantially during the exposure time. In frame-based cameras, each frame is recorded as an image in which the incident light during the exposure time is temporally integrated; therefore, the motion of the target causes strong motion blur. As a result, the blade contours and local shape information of the defective region, which are necessary for defect detection, are lost, making detection using ordinary frame-based cameras difficult. Image deblurring has been investigated for on-line surface quality inspection, but the image formation process during high-speed rotation remains a major constraint for frame-based inspection [3]. In addition, once an image has been recorded as a frame image, it is not possible to separately reconstruct only an arbitrary short time window within the exposure time or only an arbitrary rotational phase.

One possible approach to this problem is to use a high-speed camera with a short exposure time to observe targets undergoing high-speed motion. High-speed cameras are effective for observing high-speed phenomena and measuring vibration, and they have also been used for motion analysis of mechanical components [4]. However, to clearly observe defective regions with a sufficiently short exposure time, imaging conditions such as high-intensity illumination, exposure time, focus, and imaging timing must be strictly controlled. Furthermore, because high-speed cameras acquire full-frame images of all pixels at high frequency, they require dedicated equipment capable of high-speed imaging as well as large-capacity recording, transfer, and processing environments. Therefore, when long-term continuous monitoring or deployment at multiple locations is considered, they impose substantial operational constraints.

Against this background, this study focuses on event cameras. An event camera is a sensor that, instead of reading out all pixels at fixed intervals as in conventional frame-based cameras, outputs the pixel position, timestamp, and polarity as asynchronous events at the moment when the luminance change at each pixel exceeds a threshold [5]. Owing to this mechanism, event cameras have characteristics such as high temporal resolution on the order of microseconds, high dynamic range, low latency, and low data volume, making them suitable for observing objects in high-speed motion [6]. Event-based vision has also been applied to related but different tasks, including drone-propeller detection [7] and annotation-based surface-defect detection on aero-engine blades [8]. Recent studies have further explored event cameras for contactless machinery fault diagnosis and rotating-machine condition monitoring [9,10,11]. Event cameras have also been applied to industrial surface defect detection in dynamic scenes [12]. However, the output of an event camera is a temporally and spatially sparse asynchronous event stream, making it difficult to directly apply conventional frame-based image processing or general image recognition models. In particular, in defect detection for high-speed rotating propellers, events do not directly represent the object shape itself. Rather, they are generated depending on luminance changes caused by blade motion, the positional relationship with the light source, shadows, luminance differences from the background, and the rotational phase. Therefore, even for blades with the same shape, the event distribution changes depending on their position during rotation.

In this study, we investigate a sensing and analysis framework that combines an event camera and machine learning to classify normal and defective propellers rotating at high speed, crop candidate defect-related regions, and observe defect-related temporal changes. In the proposed method, asynchronous event streams acquired by the event camera are accumulated over a fixed time window to generate positive–negative (PN) composite event accumulation images, in which the distributions of positive and negative events are represented by hue and value components in the hue-saturation-value (HSV) color space. This image representation preserves not only the event occurrence locations but also the polarity of luminance changes and the total event count in a single image. Subsequently, binary classification of normal and defective propellers is performed using ResNet-18, which is widely used for image classification [13].

Furthermore, in this study, Gradient-weighted Class Activation Mapping (Grad-CAM) is used as a post-hoc visualization method to examine whether class-discriminative regions for the defective class overlap with the known defective regions [14]. Event accumulation images of high-speed rotating propellers include factors other than defects, such as imaging position shifts, individual differences among propellers, illumination conditions, shadows, and background events. Therefore, the classifier may use features unrelated to defects. To address this issue, Grad-CAM is computed for the defective class, and whether high-response regions correspond to the actual defective regions is examined. In addition, candidate defect-related regions are cropped as local regions centered on the maximum response point of Grad-CAM. Moreover, this study uses the high temporal resolution of the event camera to observe the process by which a defect progresses during rotation. Specifically, normalized cross-correlation is calculated for a sequence of event accumulation images generated at short time intervals, and the blade-passing period is estimated [15]. The rotation period is then calculated from the estimated blade-passing period, and event accumulation images are generated at the timings when the same defective blade appears at the same rotational phase. This enables time-series observation of the defective region expanding over time during rotation without stopping the propeller.

In the experiments, a three-blade propeller with a diameter of 65 mm was rotated, and both defective and normal propellers were observed using an event camera. The results suggested that normal and defective propellers could be distinguished under the tested high-speed rotation condition by using PN composite event accumulation images. Visualization using Grad-CAM showed that the high-response regions corresponded to the actual defective regions, suggesting that local event patterns near the defective regions contributed to the defective-class decisions. Furthermore, extraction of candidate defect regions based on the maximum response point of Grad-CAM showed that local images containing the defective regions could be automatically obtained. In addition, by using event accumulation images synchronized with the rotation period, the process by which a defect expanded during rotation was continuously observed at the same rotational phase, and a distance-transform-based pixel count was tracked as an image-domain indicator of defect progression. These results support the feasibility of the proposed method for classifying normal and defective propellers, cropping candidate defect-related regions, and tracking an image-domain indicator associated with defect progression without stopping high-speed rotating propellers under the tested conditions. This method may also provide a basis for future applications in in-operation inspection and predictive maintenance of industrial equipment with rotating-blade systems, such as fans, turbines, and rotary tools.

## 2. Event Camera

Conventional frame-based cameras acquire the luminance values of all pixels at fixed intervals, such as every 1/30s or 1/60s, and output them as a single frame image. Therefore, when the imaged target moves substantially during the exposure time, the shape of the target is integrated over time in the image, resulting in motion blur. In particular, for targets such as propellers rotating at high speed, whose blades move substantially within the image in a short time, the blade contours and local shape information of defective regions are likely to be lost. In contrast, an event camera is a sensor that asynchronously outputs the pixel position, timestamp, and polarity of the luminance change only when the luminance change at each pixel exceeds a fixed threshold [5,6]. Each event $e_{i}$ obtained from an event camera is expressed as follows:

(1) $$e_{i}=\left(x_{i},y_{i},t_{i},p_{i}\right),$$

where $\left(x_{i},y_{i}\right)$ denotes the pixel position at which the event occurred, $t_{i}$ denotes the timestamp at which the event occurred, and $p_{i}\in \left\{+1,-1\right\}$ denotes the polarity of the luminance change. Specifically, $p_{i}=+1$ corresponds to a positive event associated with a luminance increase, whereas $p_{i}=-1$ corresponds to a negative event associated with a luminance decrease. Because event cameras output events only from pixels where luminance changes occur, almost no data are generated from stationary backgrounds or regions with no luminance change. Therefore, the data volume can be kept lower than that of frame-based cameras, which read out all pixels at fixed intervals. In addition, because each pixel operates independently and asynchronously, event cameras have high temporal resolution on the order of microseconds and can record luminance changes in rapidly moving objects with low latency. Furthermore, because event cameras output events based on relative luminance changes at each pixel, they have a high dynamic range and can easily acquire luminance changes even in environments with large differences between bright and dark regions [5,6].

However, the output of an event camera is not a regular two-dimensional array like a frame image, but a temporally and spatially sparse asynchronous event stream. Therefore, it is difficult to directly apply conventional image processing methods or general image recognition models that assume frame images as input. To exploit the characteristics of event cameras for defect detection in high-speed rotating objects, the event stream must be converted into a representation suitable for the target task. When a propeller rotating at high speed is observed using an event camera, events are generated mainly as the blade edges move relative to the background under the illumination conditions. As the blades move, the luminance increases at some pixels when a bright blade region passes through them, whereas the luminance decreases at other pixels when the background appears after the blade has passed. These luminance changes continuously generate positive and negative events in accordance with the rotation of the propeller. The event distribution obtained in this process depends strongly not only on the geometric shape of the propeller but also on the positional relationship with the light source, shadow formation, the luminance difference from the background, and the rotational phase. Therefore, even for blades with the same shape, the number, polarity, and spatial distribution of generated events vary depending on which position in the image the blade passes through.

## 3. Proposed Method

In this study, events generated while a propeller remains in high-speed rotation are used to classify normal and defective propellers, crop candidate defect-related regions, and observe defect-related temporal changes without stopping the rotating target. The output of an event camera is an asynchronous event stream consisting of pixel positions, timestamps, and polarities, and it is difficult to feed this stream directly into general image recognition models. Therefore, in the proposed method, events generated within a fixed time window are first accumulated to generate a positive–negative (PN) composite event accumulation image, in which the distributions of positive and negative events are represented as a single red-green-blue (RGB) image. The generated event accumulation image is then fed into a convolutional neural network (CNN), and binary classification of normal and defective propellers is performed.

In addition, to examine whether regions contributing to defective-class decisions correspond to defective regions, Grad-CAM is used to visualize class-discriminative regions for the defective class. Candidate defect regions are then extracted by cropping local regions centered on the maximum response point of Grad-CAM. Furthermore, to observe the process by which a defect progresses during rotation, the rotation period is estimated based on normalized cross-correlation of a sequence of event accumulation images, and event accumulation images corresponding to the same rotational phase are generated.

The following subsections describe the generation of PN composite event accumulation images, the design of the accumulation time, defect classification using a CNN, candidate defect region extraction using Grad-CAM, and defect progression observation based on event accumulation synchronized with the rotation period.

### 3.1. Generation of PN Composite Event Accumulation Images

In this study, the set of events contained in the time interval from time $t_{k}$ to $t_{k}+\Delta T$ is defined as

(2) $$E_{k}\left(\Delta T\right)=\left\{e_{i}|t_{k}\leq t_{i}<t_{k}+\Delta T\right\}.$$

Here, ΔT is the event accumulation time.

In each time window, the positive event count $P_{k}\left(x,y\right)$, corresponding to luminance increases, and the negative event count $N_{k}\left(x,y\right)$, corresponding to luminance decreases, are defined as follows:

(3) $$P_{k}\left(x,y\right)=\sum_{\begin{array}{c} e_{i}\in E_{k}\left(\Delta T\right)\\ x_{i}=x,\;y_{i}=y,\;p_{i}=+1 \end{array}}1,$$

(4) $$N_{k}\left(x,y\right)=\sum_{\begin{array}{c} e_{i}\in E_{k}\left(\Delta T\right)\\ x_{i}=x,\;y_{i}=y,\;p_{i}=-1 \end{array}}1.$$

From the obtained $P_{k}\left(x,y\right)$ and $N_{k}\left(x,y\right)$, the total event count $C_{k}\left(x,y\right)$ and the positive event ratio $R_{k}\left(x,y\right)$ are defined as follows:

(5) $$C_{k}\left(x,y\right)=P_{k}\left(x,y\right)+N_{k}\left(x,y\right),$$

(6) $$R_{k}\left(x,y\right)=\frac{P_{k}\left(x,y\right)}{P_{k}\left(x,y\right)+N_{k}\left(x,y\right)}.$$

For pixels with $C_{k}\left(x,y\right)=0$, $R_{k}\left(x,y\right)$ is not used, and black is assigned as the background.

In this study, a PN composite event accumulation image is generated by representing the total event count $C_{k}\left(x,y\right)$ as the value component and the positive event ratio $R_{k}\left(x,y\right)$ as the hue. Because the total event count varies greatly among pixels, directly using it as the value component causes pixels with high event counts to saturate and makes it difficult to visually recognize local defect-derived events appearing in regions with low event counts. Therefore, logarithmic scaling and gamma correction are applied to the total event count. Let the maximum event count in each image be

(7) $$M_{k}=\max\left(\max_{x,y}P_{k}\left(x,y\right),\;\max_{x,y}N_{k}\left(x,y\right)\right).$$

The value component $V_{k}\left(x,y\right)$ is then defined as

(8) $$V_{k}\left(x,y\right)={\left[\frac{\log\left(1+C_{k}\left(x,y\right)\right)}{\log\left(1+2M_{k}\right)}\right]}^{\gamma }.$$

Here, γ is the gamma correction coefficient. In this study, γ=0.85 was selected empirically from the tested values because it gave the highest classification accuracy under the present experimental conditions. The hue $H_{k}\left(x,y\right)$ is defined based on the positive event ratio as follows:

(9) $$H_{k}\left(x,y\right)=\left(1-R_{k}\left(x,y\right)\right)\times 0.66.$$

The saturation is fixed at 1, and $\left(H_{k},1,V_{k}\right)$ is constructed as an HSV image and then converted into an RGB image. With this setting, pixels dominated by positive events are represented in red, pixels dominated by negative events are represented in blue, and pixels in which both types of events are mixed are represented by colors ranging from green to yellow. This representation preserves not only the event occurrence locations but also the polarity of luminance changes and the total event count in a single image.

In PN composite event accumulation images, the setting of the accumulation time ΔT greatly affects the information retained in the image. When the accumulation time is too short, event overlap among blades is limited, but the number of events required for defect detection may be insufficient. In contrast, when the accumulation time is too long, the number of events increases; however, events originating from multiple blades overlap in the same image, and local information on a defect present in a single blade may be buried by events from normal blades.

Furthermore, even for blades with the same shape, the event distribution changes depending on the rotational phase and the positional relationship with the light source. Therefore, the event distribution includes not only the blade shape and the presence or absence of defects but also factors such as shadows, illumination direction, and luminance differences from the background. In addition, when events are accumulated over a time corresponding to one full rotation, the defective blade is observed over multiple rotational phases, which may emphasize events derived from the defect. Therefore, a generally suitable accumulation time cannot be uniquely determined a priori and varies depending on the rotational speed of the target object, the number of blades, the illumination conditions, and the defect location. Accordingly, in this study, defect detection performance is evaluated while systematically varying the accumulation time ΔT as an exploratory analysis of the effect of accumulation time on detection performance.

### 3.2. CNN-Based Defect Classification

Based on the generated PN composite event accumulation images, binary classification of normal and defective propellers is performed. Because the PN composite representation contains localized spatial patterns of event polarity and density, a CNN was selected as an appropriate classifier. ResNet-18 was adopted as a standard reference backbone because this study focuses on evaluating the proposed sensing and representation framework rather than optimizing the classifier architecture [13]. The input images are resized to a specified size, and the final fully connected layer of ResNet-18 pretrained on ImageNet is replaced with a two-class output layer for the normal and defective classes [16].

Let the input image be $I_{k}$ and the classifier be $f_{\theta }$. The classifier outputs a score for each class. In this study, the probabilities of the normal and defective classes are defined using the softmax function as

(10) $$q_{k}=\mathrm{softmax}(f_{\theta }\left(I_{k}\right))=\left(q_{k,0},q_{k,1}\right),$$

where $q_{k,0}$ is the probability of the normal class and $q_{k,1}$ is the probability of the defective class. The image-level estimated label ${\hat{y}}_{k}$ is obtained as follows:

(11) $${\hat{y}}_{k}=\arg\max_{c\in \{0,1\}}q_{k,c}.$$

During training, the normal class is assigned y=0 and the defective class is assigned y=1, and a class-weighted cross-entropy loss formulation is used. This formulation allows class weights to be adjusted when the numbers of normal and defective samples are imbalanced in practical application settings. The loss function is expressed as

(12) $$L=-w_{y}\log q_{k,y},$$

where $w_{y}$ is the weight for class *y*. In the experiments reported in this paper, the training data were balanced by class; therefore, the class weights were set to $w_{0}=w_{1}=1$.

Training is performed in two stages. First, the convolutional layers of ResNet-18 are frozen, and only the final fully connected layer is trained. Subsequently, all layers are made trainable, and the entire network is fine-tuned. This two-stage training enables the model to learn features adapted to PN composite event accumulation images while using the general image feature extraction capability of the pretrained model. In addition, to improve robustness to slight shifts in the imaging position and cropping position, data augmentation including image translation and scale variation is applied during training.

In practical applications, it is not necessary to make a decision based on only a single event accumulation image; rather, an integrated decision can be made from multiple images corresponding to one full rotation or multiple rotation cycles. Therefore, in this study, in addition to image-level classification, decisions are also made by integrating the classification results of multiple images. For a group of images contained in a decision interval *K*, the average defective-class probability is defined as

(13) $$S=\frac{1}{\left|K\right|}\sum_{k\in K}q_{k,1}.$$

The interval-level estimated label $\hat{Y}$ is then determined using a decision threshold η as

(14) $$\hat{Y}=\left\{\begin{array}{cc} 1 & \left(S\geq \eta \right),\\ 0 & \left(S<\eta \right). \end{array}\right.$$

The threshold was set to η=0.5 in this study.

In this study, the stability of the proposed method as a practical defect detection system is evaluated using this integrated decision based on multiple images.

### 3.3. Candidate Defect Region Extraction Using Grad-CAM

Classification using a CNN enables normal/defective decisions for the entire input image; however, it does not directly reveal which regions in the image the classifier used to make the decision. In particular, the event accumulation images used in this study include not only event distributions derived from defects but also imaging position shifts, individual differences among propellers, the positional relationship with the light source, shadows, and background events. Therefore, even when the classification accuracy is high, the classifier does not necessarily focus on the defect itself.

Therefore, in this study, Gradient-weighted Class Activation Mapping (Grad-CAM) is applied to the trained CNN to visualize the regions that contributed to the defective-class decision [14]. In Grad-CAM, let $A^{m}$ denote the feature map in the final convolutional layer of the CNN and let $s^{c}$ denote the output score for the defective class *c*. The weight $\alpha_{m}^{c}$ is calculated from the spatial average of the gradients with respect to the feature map $A^{m}$ as follows:

(15) $$\alpha_{m}^{c}=\frac{1}{Z}\sum_{i}\sum_{j}\frac{\partial s^{c}}{\partial A_{ij}^{m}},$$

where *Z* is the spatial size of the feature map. Using the obtained weights, the Grad-CAM map $L_{\mathrm{Grad}-\mathrm{CAM}}^{c}$ for the defective class is calculated as

(16) $$L_{\mathrm{Grad}-\mathrm{CAM}}^{c}=\mathrm{ReLU}\left(\sum_{m}\alpha_{m}^{c}A^{m}\right).$$

The obtained Grad-CAM map is upsampled to the input image size, normalized, and then overlaid on the PN composite event accumulation image. Here, ReLU(·) denotes the rectified linear unit. This enables visual inspection of the regions contributing to the defective-class decision.

Furthermore, in this study, Grad-CAM is also used to extract candidate defect regions. The maximum response point in the normalized Grad-CAM map is defined as

(17) $$\left(x^{*},y^{*}\right)=\arg\max_{x,y}L_{\mathrm{Grad}-\mathrm{CAM}}^{c}\left(x,y\right),$$

and this point is regarded as the candidate defect location. A local region centered on $\left(x^{*},y^{*}\right)$ is then cropped and saved as a candidate defect image. Through this process, a local image containing the defective region can be extracted from a classifier trained using only normal/defective labels for the entire image, without providing detailed supervisory information on the defect location.

### 3.4. Defect Progression Observation Using Normalized Cross-Correlation

To observe defect progression in a propeller rotating at high speed, the blade containing the defect must appear at the same rotational phase in images generated at different times. With a conventional frame-based camera, the propeller moves substantially during the exposure time, making it difficult to capture local shape changes in the defective region. In contrast, an event camera can record luminance changes with high temporal resolution, enabling event accumulation images to be generated from the acquired event stream using an arbitrary time window.

In this study, the rotation period of the propeller is estimated using normalized cross-correlation of a sequence of event accumulation images, and event accumulation images at the same rotational phase are generated based on the estimated period [15]. First, a sequence of event accumulation images $\left\{F_{0},F_{1},\ldots ,F_{K}\right\}$ is generated at a short time interval $\Delta t_{f}$. Next, the normalized cross-correlation ρ(k) between the reference image $F_{0}$ and the image $F_{k}$, which is obtained at a lag of *k* time steps, is calculated as

(18) $$\rho \left(k\right)=\frac{\sum_{x,y}\left(F_{0}\left(x,y\right)-{\bar{F}}_{0}\right)\left(F_{k}\left(x,y\right)-{\bar{F}}_{k}\right)}{\sqrt{\sum_{x,y}{\left(F_{0}\left(x,y\right)-{\bar{F}}_{0}\right)}^{2}\sum_{x,y}{\left(F_{k}\left(x,y\right)-{\bar{F}}_{k}\right)}^{2}}}.$$

Here, ${\bar{F}}_{0}$ and ${\bar{F}}_{k}$ are the mean luminance values of images $F_{0}$ and $F_{k}$, respectively. For a three-blade propeller, the event accumulation images become similar each time adjacent blades pass through the same relative position; therefore, ρ(k) has a peak at the time lag corresponding to the blade-passing period. In this study, the first local peak of ρ(k) after k=0 is denoted as $k_{b}$, which corresponds to the blade-passing period.

The blade-passing period $T_{b}$ is then expressed as

(19) $$T_{b}=k_{b}\Delta t_{f}.$$

When the number of blades is $N_{b}$, the rotation period $T_{r}$ is given by

(20) $$T_{r}=N_{b}T_{b}.$$

Because the propeller used in this study has three blades,

(21) $$T_{r}=3T_{b}.$$

Let $t_{0}$ be the reference time, τ be the offset representing the phase at which the defective blade can be readily observed, and $\Delta T_{g}$ be the accumulation time used for defect progression observation. The *m*-th event accumulation image at the same rotational phase is generated from the events contained in the following time interval:

(22) $$\left[t_{0}+mT_{r}+\tau ,\;t_{0}+mT_{r}+\tau +\Delta T_{g}\right].$$

In the image sequence generated in this manner, the blade containing the defect appears at almost the same position in the image. Therefore, images at different times can be compared, and defect progression can be quantitatively evaluated.

First, for the defect-region image cropped using Grad-CAM, a 20×20-pixel region of interest (ROI) is set at the center, and non-black pixels are extracted as an event point set. Next, the distance from this point set to each pixel is obtained using a distance transform. The distance transform is a process that calculates the distance from each pixel to the nearest event-point pixel. Specifically, the distance value is 0 at locations where event-point pixels exist, and larger distance values are assigned to blank regions farther from the event point set. In this study, regions close to the event-point pixels are treated as normal event distributions, whereas blank regions far from the point set are treated as defective regions. Let $B_{m}\left(u,v\right)$ denote a binary image in which event-point pixels are assigned 1. The distance image $D_{m}\left(x,y\right)$ is defined as follows:

(23) $$D_{m}\left(x,y\right)=\min_{\begin{array}{c} \left(u,v\right):\;B_{m}\left(u,v\right)=1 \end{array}}\sqrt{{\left(x-u\right)}^{2}+{\left(y-v\right)}^{2}}.$$

Here, (u,v) denotes the coordinates of an event-point pixel. Therefore, $D_{m}\left(x,y\right)$ represents the Euclidean distance from pixel (x,y) to the nearest event point. In the implementation, the distanceTransform function of the Open Source Computer Vision Library (OpenCV, version 4.12.0) is used [17]. Because distanceTransform calculates the distance to zero-valued pixels, an image in which the event-point pixels are set to 0 and all other pixels are set to 255 is used as the input, thereby obtaining the distance from each pixel to the nearest event point. The $L_{2}$ distance is used. This enables pixels whose distance values in the ROI are greater than or equal to a threshold to be extracted as candidate defect-related regions. The number of extracted pixels is then used as an image-domain indicator associated with defect progression. This pixel count is treated as a two-dimensional image-domain indicator, because accurate conversion to physical surface area would require camera calibration and three-dimensional information on the curved propeller blade.

## 4. Experiments

**Figure 1:**
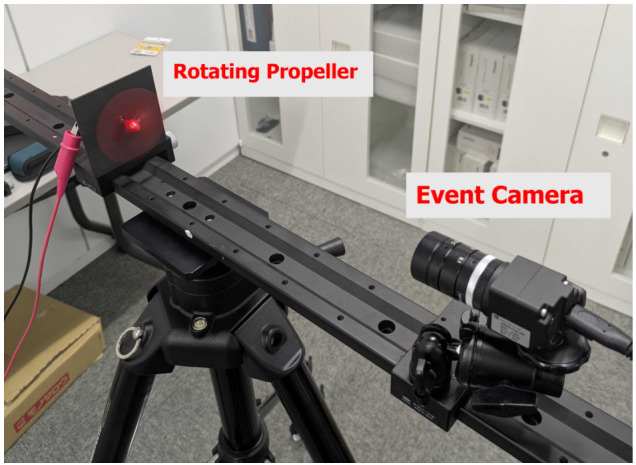
Experimental setup for event-camera-based observation of a high-speed rotating propeller. The main components are labeled in the figure: the rotating propeller, which served as the inspection target, and the event camera. The event camera and the propeller were fixed on the same rail so that the camera faced the propeller from the front and the rotating propeller remained within the field of view throughout the experiment.

**Figure 2:**
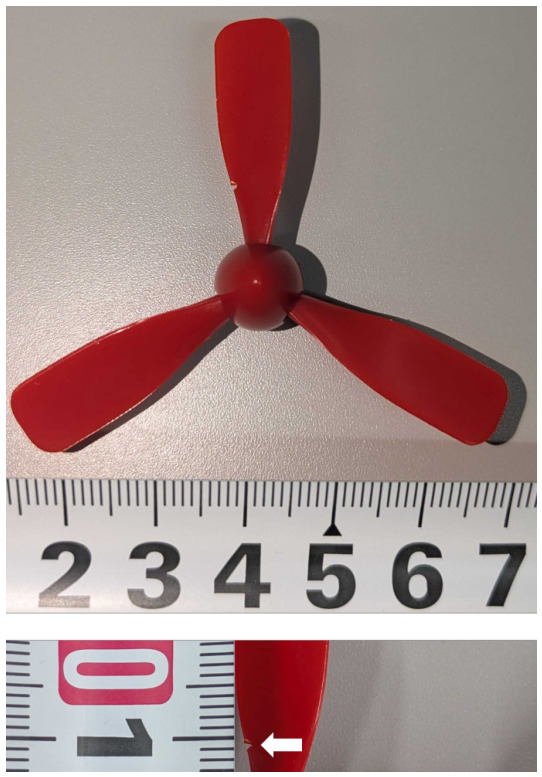
Three-blade propeller used in the experiments. The propeller had an outer diameter of 65 mm, and artificial defects were introduced into one blade for defective samples.

The defect detection system used in this paper consists of a target region containing the rotating propeller and an event camera fixed at a position that captures the target region within its field of view. Figure 1 shows the experimental setup. The experiments were conducted under standard HFE inverter fluorescent lamps installed on the laboratory ceiling, and no additional special illumination equipment was used. The event camera was a SilkyEvCam HD (CenturyArks Co., Ltd., Tokyo, Japan) equipped with an IMX636 HD event-based vision sensor jointly developed by Sony Semiconductor Solutions Corporation (Atsugi, Japan) and Prophesee SA (Paris, France), and the lens was an M0814-MP2 8 mm lens (Computar, CBC Co., Ltd., Tokyo, Japan). The observed target was a three-blade propeller with an outer diameter of 65 mm, made of plastic. Figure 2 shows the propellers used in the experiments. The event camera was operated using the default acquisition configuration of the software development kit (SDK; Metavision Studio, version 5.1.1, Prophesee SA, Paris, France). For the IMX636 sensor, the Metavision Studio user-tunable bias values are defined as offsets from the factory-trimmed defaults; in this experiment, these offsets were kept at their default values: bias_diff = 0, bias_diff_on = 0, bias_diff_off = 0, bias_fo = 0, bias_hpf = 0, and bias_refr = 0. No manual tuning was applied to camera-level event-generation, ROI, anti-flicker, event-trail filtering, or event-rate-control settings in Metavision Studio.

Event accumulation-image generation, CNN training and evaluation, Grad-CAM analysis, and distance-transform-based analysis were performed on a workstation equipped with an AMD Ryzen Threadripper PRO 3995WX 64-core CPU, an NVIDIA RTX A6000 GPU, and 224 GB RAM, running Windows 11.

### 4.1. Dataset

In this experiment, 10 propellers were prepared: five defect-free propellers as normal data and five propellers as defective data, each of which had an artificial defect introduced into one blade of a three-blade propeller. For the defective propellers, defects were created manually by cutting out approximately 1 mm of one blade at comparable positions and with comparable sizes.

These propellers were rotated at approximately 862rpm and recorded using the event camera. Each propeller was recorded five times, resulting in 25 trials for normal propellers and 25 trials for defective propellers, for a total of 50 recording trials. For each recording, the PN composite event accumulation images described in Section 3 were generated, and 5000 images were created per recording. Therefore, the dataset used in this experiment consisted of 125,000 normal images and 125,000 defective images, for a total of 250,000 event accumulation images. To reduce the influence of background events outside the propeller, a 300×300-pixel region of interest containing the propeller was cropped from each generated image and resized to 224×224 pixels when input to the CNN. In the classification experiment, each recording trial was managed as a single data folder, and the dataset was constructed from multiple trials of normal and defective propellers.

For evaluation, a propeller-instance-level holdout split was used instead of an image-level random split. Specifically, for each seed, one normal propeller and one defective propeller were selected as test propellers, and all recording folders and all event accumulation images generated from these two physical propeller instances were used only as test data and excluded from the training and validation data. The remaining folders were used to construct the training and validation data, which were split into 70%/30% subsets for each class. This split prevented similar images generated from the same trial, as well as images from different trials of the same physical propeller, from being included in both the training and test data. Therefore, the evaluation examined generalization to unseen physical propeller instances and recording trials within the same 65 mm plastic three-blade propeller type and the defect condition examined in this study.

The classification experiments were repeated five times using different random seeds, and the reported accuracy represents the mean over these five runs.

The classifier was the ResNet-18 pretrained on ImageNet described in Section 3.2 [13,16]. First, only the output layer was trained for 3 epochs. Subsequently, all layers were fine-tuned for 10 epochs. The learning rate was $1.0\times 10^{-3}$ during output-layer training and $1.0\times 10^{-4}$ during fine-tuning, and AdamW was used as the optimization method [18].

### 4.2. Defect Detection Performance

In this section, the effect of event accumulation time on defect detection performance was first examined by systematically varying the accumulation time ΔT. Specifically, ΔT was varied from 100 μs to 5000 μs in increments of 100 μs, and event accumulation image generation, training, and test evaluation were performed for each accumulation time. This sweep was conducted as an exploratory analysis of the sensitivity to ΔT, rather than as an independent hyperparameter-selection procedure.

To clarify the contribution of the PN composite event accumulation image, a simple event-histogram baseline was also evaluated using the same CNN backbone and the same propeller-instance-level holdout split. In the event-histogram representation, positive and negative events within the same accumulation window were counted together at each pixel and used as a polarity-independent event-count image. The same training protocol was used for the PN composite representation and the event-histogram baseline.

**Figure 3:**
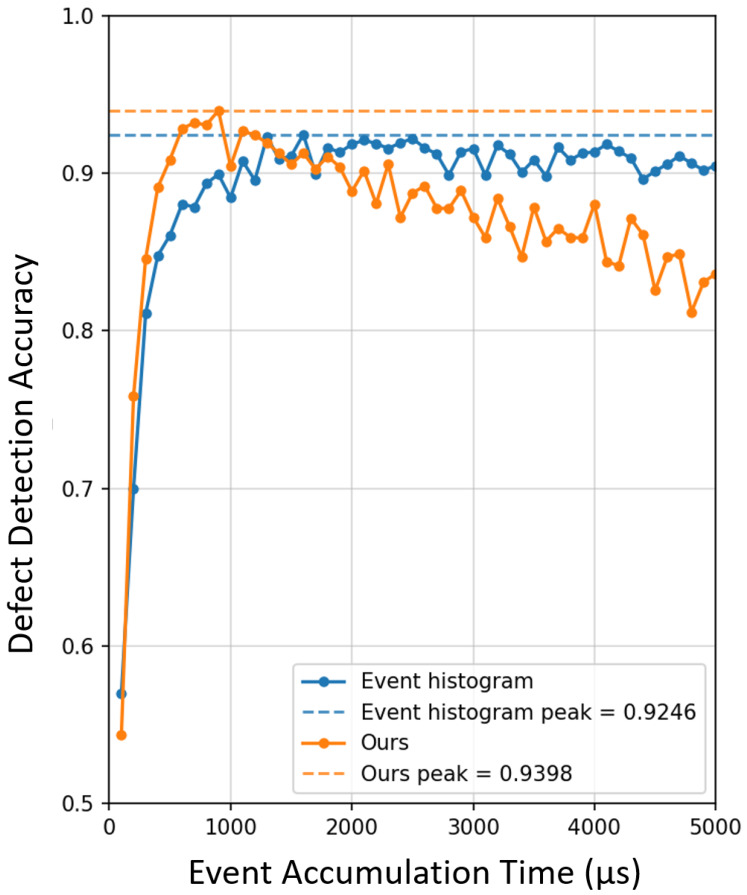
Comparison of defect detection accuracy between the PN composite event accumulation image and the event-histogram baseline as a function of event accumulation time. The event-histogram baseline counts positive and negative events together without using polarity information. Dashed lines indicate the peak mean accuracies of each representation.

**Figure 4:**
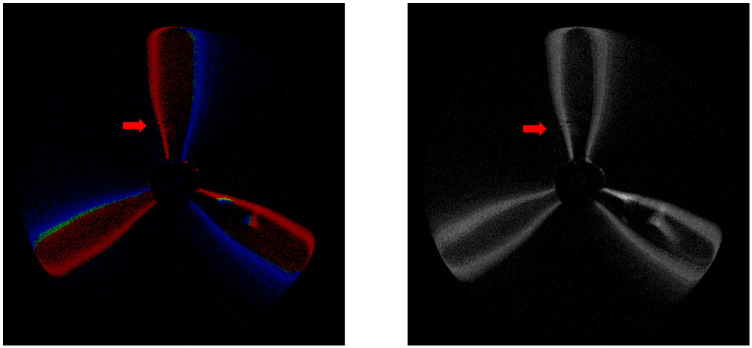
Representative examples of the PN composite event accumulation image at 900 μs and the event-histogram image at 1600 μs. (**Left**): PN composite event accumulation image at 900 μs, where the PN composite representation achieved its peak mean accuracy. (**Right**): polarity-independent event-histogram image at 1600 μs, where the event-histogram baseline achieved its peak mean accuracy. Red arrows indicate the known defective region.

Figure 3 shows the defect detection accuracy when the accumulation time was varied. As shown in Figure 3, the PN composite representation achieved a higher peak mean accuracy than the event-histogram baseline under the present experimental conditions. The peak mean accuracy of the PN composite representation was 0.9398, whereas that of the event histogram was 0.9246. The standard deviation of the PN composite representation at the 900 μs accumulation time was 0.00585 across five runs with different random seeds. This result suggests that encoding polarity-dependent event patterns was effective, particularly at short accumulation times. Figure 4 shows representative examples of the PN composite event accumulation image at 900 μs and the event-histogram image at 1600 μs, corresponding to the peak mean accuracies of the PN composite representation and the event-histogram baseline, respectively.

One possible reason for this trend is that, within a short accumulation window, the spatial separation of positive and negative events generated along the moving blade edges is preserved, and local polarity patterns caused by defects can remain distinguishable. In contrast, when the accumulation time is increased, positive and negative event trajectories can overlap at or near the same pixels, increasing polarity mixing in the positive-event ratio. As a result, the discriminability of the hue contrast encoding event polarity can decrease. In addition, local defect-related events may be diluted within the longer swept trajectories generated by the overall rotational motion of the propeller.

On the other hand, the event histogram functions as a time-averaged event-density map without using polarity information. Therefore, after a sufficient number of events has been accumulated, its performance remains relatively stable even when the accumulation time increases. However, this representation cannot exploit the polarity-dependent features that contributed to the higher peak performance of the PN composite representation. This comparison clarifies the effectiveness of the proposed representation relative to a simple event-count baseline under the present dataset and experimental conditions.

In practical applications, it is not necessary to determine the presence or absence of a defect from only a single image; rather, an integrated decision can be made using multiple event accumulation images corresponding to one full rotation or multiple rotation cycles. Therefore, in this study, the classification results of multiple images corresponding to one full rotation were integrated to make a rotation-level normal/defective decision. Specifically, as defined in Section 3.2, the average defective-class probability was calculated for the group of images included in one full rotation, and normal/defective classification was performed based on this value. As a result, no misclassifications were observed for the integrated decision over one full rotation under the present dataset.

This result suggests that integrating observations from multiple phases improved the stability of normal/defective decisions in the present dataset compared with using only a single image. In particular, because propeller defects are repeatedly observed every rotation, the high-temporal-resolution data obtained by the event camera provide multiple decision opportunities within a short period. This can help improve robustness against temporary event loss and illumination-dependent variations.

### 4.3. Visualization and Candidate Defect-Region Extraction Using Grad-CAM

The previous section showed that defects present in a propeller rotating at high speed can be detected under the tested conditions using PN composite event accumulation images. However, even when the classification accuracy is high, the classifier does not necessarily focus on the defect itself. Event accumulation images include not only event distributions derived from defects but also the effects of imaging position shifts, individual differences among propellers, the positional relationship with the light source, shadows, and background events. Therefore, the possibility that the classifier uses features not directly related to defects to make normal/defective decisions cannot be excluded.

Accordingly, in this experiment, Grad-CAM was applied to the trained ResNet-18 to visualize the regions that contributed to the defective-class decision. Grad-CAM was calculated for the final convolutional block of ResNet-18, and the obtained heatmap was overlaid on the input PN composite event accumulation image. In addition, the maximum response point in the Grad-CAM map was regarded as the candidate defect location, and a 64×64-pixel local region centered on this point was cropped.

**Figure 5:**
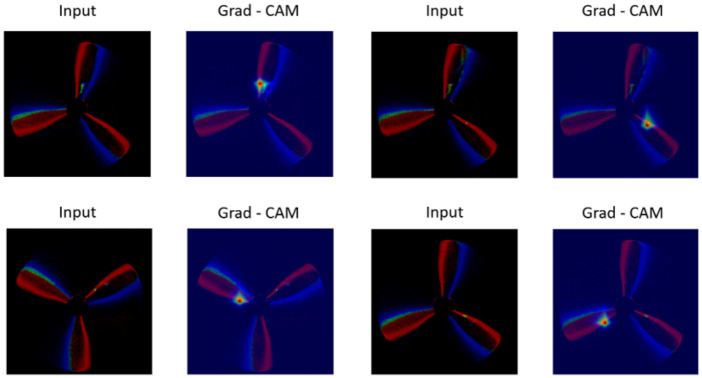
Examples of Grad-CAM visualization for defective propellers. PN composite event accumulation images and the corresponding Grad-CAM overlay results are shown.

**Figure 6:**
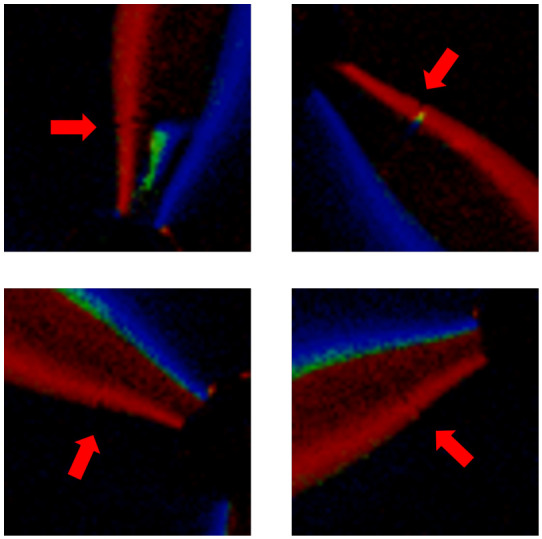
Examples of candidate defect-region images cropped around the maximum response point of Grad-CAM. The arrows indicate defective regions included in the cropped local regions.

Figure 5 shows examples of a PN composite event accumulation image and the corresponding Grad-CAM overlay result for a defective propeller, and Figure 6 shows examples of candidate defect-region images cropped around the maximum response point of Grad-CAM. The high-response regions in Grad-CAM corresponded well to the defective regions on the propeller blades.

For the Grad-CAM-based candidate-region analysis, 5000 images were extracted from defective-propeller images that were correctly classified as defective by the trained classifier. For each selected correctly classified defective image, a 64×64-pixel local image was cropped around the maximum response point of Grad-CAM. The result was counted as successful when the known defective region was included in the cropped local image.

In this rule-based visual inclusion evaluation, the known defective region was included in the cropped local image for all 5000 correctly classified defective images used in the Grad-CAM analysis, resulting in an inclusion rate of 100%. This result indicates that, for the correctly classified defective images inspected, the Grad-CAM maximum-response point provided a candidate region that included the known defect.

Defective images that were misclassified as normal were examined separately with respect to their rotational phase and acquisition time. No clear concentration of misclassifications at a specific rotational phase or a specific time interval was observed. Therefore, under the tested conditions, the misclassifications appeared to be sporadic and may be attributable to transient factors such as noise, shadows, or illumination-dependent event variations, rather than to a systematic failure at a specific rotational phase. The classifier used in this experiment was trained using only normal/defective labels assigned to the entire image as supervisory information, without using annotations for defect locations. These results suggest that Grad-CAM can be used to propose candidate defect-related regions from image-level labels under the tested conditions.

### 4.4. Observation of Defect Progression Behavior in a Rotating Propeller

In this experiment, an initial defect larger than the small defects used in the previous sections was introduced into a propeller blade, and the process by which the defect expanded during high-speed rotation at 2610rpm was recorded using the event camera. In addition, based on the period estimation described in Section 3.4, event accumulation images were generated at the timings when the blade containing the defect appeared at the same rotational phase.

**Figure 7:**
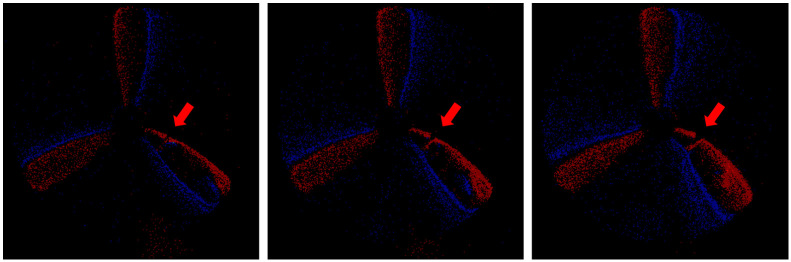
Time-series observation of defect-related event-pattern changes at approximately the same rotational phase.

**Figure 8:**
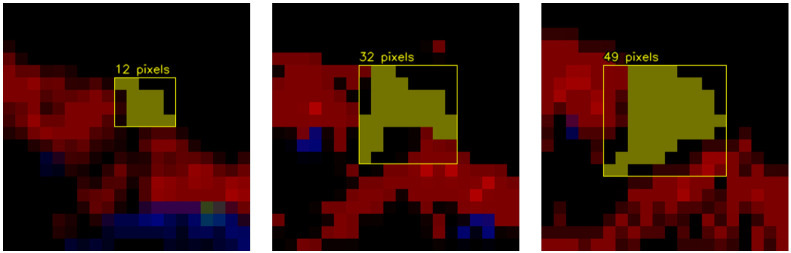
Distance-transform-based pixel-count indicator associated with defect progression in the progression experiment. The extracted pixel count increased over time, and the defective blade fractured after the measured pixel-count indicator had increased.

As shown in Figure 7, the local event distribution corresponding to the defective region changed over time, and defect-related event-pattern changes were observed. In addition, Figure 8 shows that the distance-transform-based pixel count increased over time, providing an image-domain indicator associated with defect progression during rotation. In this progression experiment, the defective blade fractured after the measured pixel-count indicator had increased.

This result suggests that event data acquired while the propeller remained in high-speed rotation can be used to observe defect-related event-pattern changes under the tested conditions. This progression experiment was conducted on one propeller and one failure event. Therefore, the repeatability of the pixel-count trend and the relationship between the pixel-count indicator and fracture timing should be evaluated in future experiments using multiple propellers and multiple progression or failure events. The present result indicates the possibility of image-domain observation of defect-related changes during high-speed rotation.

## 5. Conclusions

Defect detection for high-speed rotating propellers can be approached using frame-based cameras or high-speed cameras. However, standard frame-based cameras are prone to losing local shape information in defective regions because of motion blur, whereas high-speed cameras face constraints such as equipment cost and the need for high-intensity illumination. In this study, we investigated a method that exploits the high temporal resolution and asynchronous operation of an event camera, represents events generated by a propeller during high-speed rotation as PN composite event accumulation images, and classifies normal and defective propellers using a CNN. The experiments suggested that normal and defective propellers could be distinguished from a single event accumulation image under the tested conditions. Furthermore, by integrating multiple images corresponding to one full rotation, no misclassifications were observed under the present dataset.

Grad-CAM was also used as a post-hoc visualization method, and the high-response regions visually overlapped with the known defective regions in the inspected samples. This result suggests that local event patterns near the defective regions may have been associated with the defective-class decisions. Furthermore, candidate defect-related regions were cropped using the maximum response point of Grad-CAM, and the known defective regions were included in the cropped local images in the inspected cases. In addition, event accumulation images at the same rotational phase were generated based on the rotation period estimated using normalized cross-correlation, and the process by which a defect expanded during rotation was observed. Moreover, using the distance transform, empty regions far from the event point set were extracted as defect regions, and the distance-transform-based pixel count was tracked as an image-domain indicator of defect progression. These results support the feasibility of the proposed method for classifying normal and defective propellers, cropping candidate defect-related regions, and tracking an image-domain indicator associated with defect progression without stopping high-speed rotating propellers under the tested conditions. Future work will investigate applicability to different propeller geometries, materials, defect types, including naturally occurring defects, rotational speeds, illumination conditions, camera settings, and real operating environments, as well as extension to real-time processing. Comparison with lightweight and more recent classifier architectures on larger and more diverse datasets also remains an important direction for future work.

## Data Availability

The data and source code supporting the findings of this study are available from the corresponding author upon reasonable request.
